# An RTCA-based assay as an innovative approach for thermal inactivation studies of hepatitis A virus

**DOI:** 10.1128/aem.01822-25

**Published:** 2026-02-10

**Authors:** Audrey Fraisse, Laurent Guillier, Sylvie Perelle, Sandra Martin-Latil

**Affiliations:** 1 Laboratory for food safety, Université Paris-Est, ANSES 133617 , Maisons-Alfort, France; 2 Risk Assessment Department, ANSES, Maisons-Alfort, France; 3 UMR VIROLOGIE, ANSES, INRAE, Ecole Nationale Vétérinaire d’Alfort, Université Paris Est 133617 , Maisons-Alfort, France; Centers for Disease Control and Prevention, Atlanta, Georgia, USA

**Keywords:** food safety, predictive model, RTCA assay, heat inactivation, hepatitis A virus

## Abstract

Hepatitis A virus (HAV) is responsible for acute viral hepatitis worldwide, mainly transmitted via the fecal-oral route either through direct contact or by consuming contaminated food. Thermal inactivation is widely applied in the food industry to inactivate viruses and ensure the safety of food products. The effectiveness of inactivation treatments depends on the initial viral load and the sensitivity of the method quantifying infectious viruses. This study aimed to assess the real-time cell analysis (RTCA) assay in the framework of heat inactivation studies, compare its suitability to the traditional PFU assay and molecular-based methods (RT-qPCR, integrity-RT-qPCR), and model thermal inactivation kinetics. Our results showed that the RTCA assay is a suitable method for quantifying the decrease in infectious HAV as the time-temperature scale increases. No more infectious virus was detected after treatment at 37°C, 50°C, 65°C, 72°C, and 80°C, lasting 9 days, 24 h, 10 min, 5 min, and 2 min, respectively. The Geeraerd model was identified as the best fit to describe HAV thermal inactivation kinetics. The *D* values, corresponding to the time required to achieve a 1 log_10_ reduction in HAV titer*,* were comparable between both cell-culture-based methods across all tested temperatures. In contrast, molecular methods yielded significantly higher *D* values. Kinetic parameters were subsequently validated, confirming that the isothermal-derived parameters can reliably predict viral inactivation under dynamic thermal conditions. This study demonstrates that the RTCA assay provides a powerful tool for modeling HAV thermal inactivation that can offer valuable guidance for implementing effective food safety strategies.

**IMPORTANCE:**

There has been a significant increase in viral foodborne outbreaks following the consumption of raw or minimally processed foods. Thermal treatments may ensure food safety, with efficacy depending on viral load and method sensitivity. In this study, we present a powerful real-time cell analysis assay that advances our analytical ability to quantify infectious hepatitis A virus. By providing kinetic parameters comparable to those obtained with the traditional infectious titration method, this high-throughput assay overcomes its limitations to conduct viral inactivation studies on a larger scale. In addition, the successful validation of kinetic parameters confirms that isothermal-derived parameters can reliably predict viral inactivation under dynamic thermal conditions. This offers a reliable and essential tool to strengthen food safety measures to be applied in the food industry.

## INTRODUCTION

Hepatitis A virus (HAV), which belongs to the genus *Hepatovirus* within the family *Picornaviridae*, is a non-enveloped virus containing a positive-sense single-stranded RNA genome (1). Hepatitis A is the most common form of acute viral hepatitis worldwide. Most HAV infections acquired during early childhood are asymptomatic, whereas adults exhibit symptoms such as fever, loss of appetite, diarrhea, nausea, dark-colored urine, and jaundice (2, 3). The HAV infection rate is strongly associated with socioeconomic factors and sanitation conditions. Even if HAV infection is less common in countries with a high standard of hygiene, it can lead to a more severe disease outcome due to a later infection in life (4). HAV transmission mainly occurs via the fecal-oral route through person-to-person contact or through the ingestion of contaminated food and water (4). In high-income countries, besides the risk factor of traveling to countries with high or intermediate HAV endemicity (5–8), the foodborne outbreaks are increasingly reported as being associated with food imports from endemic countries. Indeed, large foodborne hepatitis A outbreaks were reported in the past 10 years to be associated with consumption of foodstuffs distributed in different European countries, Australia, and in the United States (USA). For example, a prolonged outbreak that occurred between January 2013 and May 2014 in 11 European countries (Bulgaria, Denmark, France, Germany, Ireland, Italy, the Netherlands, Norway, Poland, Sweden, and the United Kingdom), affecting nearly 1,500 patients, was linked to the consumption of frozen mixed berries (9). The food vehicles most frequently implicated in viral foodborne outbreaks include raw and minimally processed foods, particularly bivalve molluscan shellfish (e.g*.*, oysters, mussels), fresh vegetables (e.g., soft fruits, salads), and drinking water (10, 11).

The use of efficient processes that inactivate or remove viruses from water and food is crucial to control the transmission of viruses and reduce the burden of viral foodborne illnesses. According to the food matrices concerned with the viral hazard, food industries implement diverse technological treatments to ensure food safety. Several food-processing treatments, such as washing and freezing, appear to be inadequate for inactivating or removing viruses, which may remain infectious well beyond the food product’s shelf life (12). On the contrary, heat and chlorine are among the most effective measures for the inactivation of HAV and are widely applied in the food industry. However, relatively high temperatures (≥65°C) are required to achieve complete HAV inactivation. Such heat treatments cannot always be applied to certain food products, as they may alter their sensory and nutritional qualities. Similarly, chlorine is only effective against HAV at relatively high concentrations (20–200 mg/L), depending on the matrix and contact time, which may limit its practical application in some food products (13–15). Viral inactivation is always measured as a log reduction factor, and food industries have to ensure at least 4.0-log removal of pathogenic viruses by using technological treatments (16, 17). Nevertheless, their effectiveness should not be reduced to these reduction factors, as it also depends on the viral load in the starting material and the sensitivity of the assay used.

Predictive microbiology applied to virology provides an essential framework to translate experimental inactivation data into quantitative parameters (*D* and *Z* values) that can be used to design and optimize thermal processes. By linking temperature to inactivation kinetics, this approach offers a practical tool to propose and justify effective control measures in food processing (18, 19).

The assessment of treatment efficiency relies on the use of detection methods quantifying viral infectious particles. Molecular-based methods are not suitable for inactivation studies because of their inability to discriminate between the infectious and noninfectious viral particles, leading to an underestimation of treatment efficiency (20, 21). In the absence of a cell-culture-based method, capsid-integrity-RT-qPCR assays were developed by using dyes such as ethidium monoazide (EMA) or propidium monoazide. They were successfully applied to detect genomes from intact particles of enterovirus, rotavirus, murine norovirus, HAV, and norovirus following heat treatment, thus providing a closer approximation to infectivity (22–25). These dyes selectively penetrate viral particles with damaged capsids and covalently bind to the viral RNA upon light activation, thereby hampering its amplification during RT-qPCR. In contrast, RNA encapsidated within intact viral particles remains protected from dye binding and thus can be efficiently amplified by RT-qPCR. Nevertheless, only high temperatures could sufficiently damage viral particles to enable the efficiency of the capsid-integrity-RT-qPCR assay in estimating viral infectivity (24). Traditional cell culture-based methods, such as plaque assay or TCID50, may be used in the inactivation studies and analyses of virus reduction. Given that both cell culture-based methods used for titration of the adapted strains of HAV (HM175/18f) are time-consuming and labor-intensive, we previously developed a real-time cell analysis (RTCA) assay using cell impedance as a readout of HAV-induced cell death (26).

The overall aims of the study were to evaluate the implementation of the recently developed RTCA assay for heat inactivation studies and then to compare its suitability to other available quantification methods: PFU assay, integrity-RT-qPCR, and RT-qPCR. The effect of temperature on HAV was examined, and thermal inactivation kinetics were modeled using linear regression analysis to determine *D* and *Z* values for each quantification method. These kinetic parameters were subsequently validated using dynamic temperature conditions and predictive modeling through the Bioinactivation web application.

## RESULTS

### Standard curve for the quantification of infectious HAV by RTCA assay

**Fig 1:**
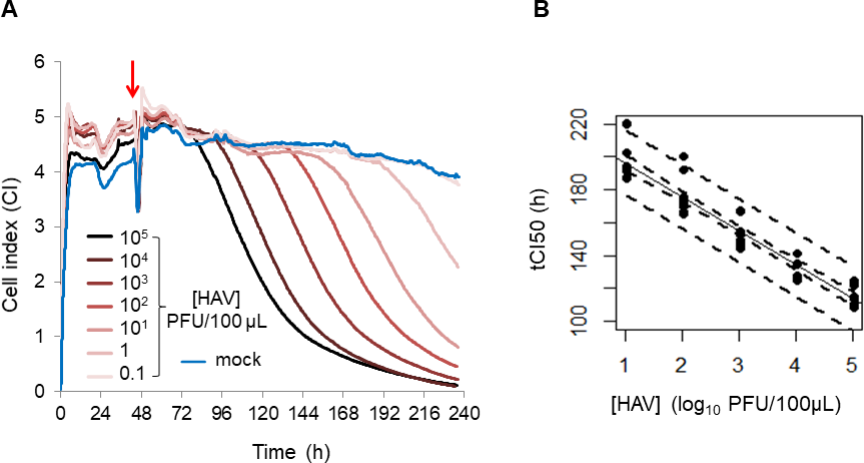
Quantification of infectious HAV using RTCA-based assay. (**A**) Kinetic curves of CI values in FRhK-4 cells uninfected (blue, “mock”: cells treated with virus-free infection medium) or infected with different concentrations of HAV (from 10^5^ PFU/100 µL [black] to 0.1 PFU/100 µL [pink]). The red arrow indicates the time at which HAV was added to sub-confluent monolayers of FRhK-4 cells. (**B**) Calibration curve between tCI_50_ and log_10_ of HAV concentration. Values of tCI_50_ are presented as the time (hours post-seeding) at which a 50% decrease in CI values is observed. The straight line corresponds to the linear regression, and the dotted lines indicate the prediction interval at 95% and the confidence interval of the regression line.

To use the RTCA assay as a titration method for the quantification of infectious HAV following thermal inactivation treatments, a standard curve was first established. FRhK-4 cells were infected with serial 10-fold dilutions of HAV stock (HAV concentrations ranging from 10^0^ to 10^6^ PFU per mL) and Cell Index (CI) values of mock- and HAV-infected cells were recorded for 8 days in real time with the xCELLigence system (Fig. 1).

Kinetic curves of CI showed that the cellular impedance of FRhK-4 cells was stabilized in mock-infected cells between 3.91 ± 0.50 and 4.50 ± 0.35 over the full length of infection, whereas a CI drop was observed in cells infected with HAV suspensions equal to or higher than 1 PFU per 100 µL (Fig. 1A). Since the time to reach CI_50_ (tCI_50_) was dependent on the HAV concentration, an inverse linear relation could be established between both parameters (Fig. 1B). The tCI_50_ increased by around 20 h, while the HAV quantities decreased by 1 log_10_.

Thus, this standard curve was further used for the titration of infectious HAV in samples with unknown viral concentrations. The sensitivity of the RTCA assay was 1 PFU per well, and the RTCA assay covered a range of 5 log_10_ PFU of HAV.

### Monitoring of HAV thermal inactivation by RTCA assay

**Fig 2:**
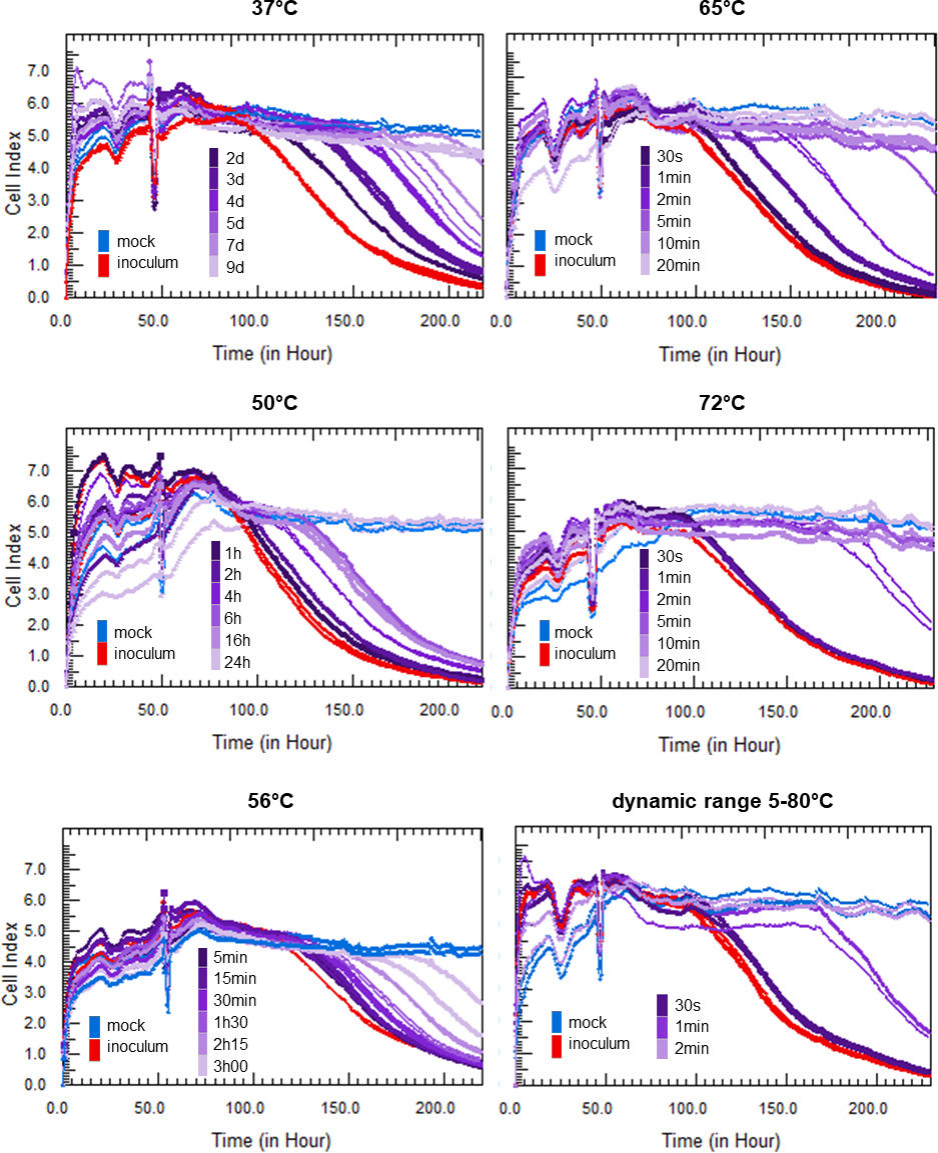
Real-time impedance analysis of mock-infected (blue) and HAV-infected (red: inoculum; purple: heat-treated HAV) FRhK-4 cells at different incubation temperatures (37°C, 50°C, 56°C, 65°C, and 72°C and a dynamic range of 5°C–80°C). The incubation time is represented by a color gradient, with the longest times represented by the lightest colors. Representative data from one of three independent experiments are shown.

To evaluate the use of the RTCA assay in the framework of HAV thermal inactivation studies, the CI values of mock-infected cells and cells infected with either untreated HAV inoculum or heat-treated samples at various temperatures (37°C, 50°C, 56°C, 65°C, 72°C, or 80°C) were measured in real time using the xCELLigence system. Kinetics curves of CI values are displayed in Fig. 2 for every temperature according to the duration of heat treatment.

Kinetics of CI values showed that CI remained constant in mock-infected cells, whereas a CI drop occurred in FRhK-4 cells infected with HAV inoculum (e.g., titered by Plaque assay at 2.16 × 10^4^ PFU/100 µL; *n* = 18). The mean of tCI_50_ values obtained for FRhK-4 cells infected with HAV inocula was 129.7 ± 1.4 h post-seeding (p.s.), corresponding to 82.6 ± 1.5 h post-infection (p.i.). The previously established calibration curve allowed the quantification of infectious HAV inoculum at 2.22 × 10^4^ PFU/100 µL (*n* = 18). When cells were infected with heat-treated samples, tCI_50_ values increased along with the duration of heat treatment at constant temperature until there was no longer any decrease in CI. Figure 2 illustrates representative data from one of three independent experiments. Nevertheless, inter-assay variability did not exceed a standard error of 0.99 h (see Table 1). These increases in tCI_50_ values showed that the quantity of infectious viruses decreased over time of heat treatment. As an example, the average of tCI_50_ values obtained for HAV treated at 56°C increased from 141.6 ± 2.9 h p.s. to 152.8 ± 2.2 h p.s. when the duration of heat treatment increased from 5 to 30 min, meaning that the amount of infectious viruses decreased from 3.8 ± 0.1 log_10_ PFU to 3.2 ± 0.1 log_10_ PFU. In the same manner, increasing the temperature of treatment by keeping the same duration of heat treatment led to an increase in tCI_50_ values, and thus to a decrease in infectious viruses. As an example, HAV treated for 1 min at 65°C and 72°C showed a mean tCI_50_ value of 142.0 ± 2.4 h p.s. and 161.8 ± 6.9 h p.s., respectively, corresponding to a decrease in the amount of infectious viruses from 3.8 ± 0.1 log_10_ PFU to 2.8 ± 0.3 log_10_ PFU.

**TABLE 1:** Analysis of heat-treated HAV samples by RTCA assay

37°C		tCI_50_ (h)^*a*^	65°C	tCI_50_ (h)
Mock		ND*^b^* (0/6)	Mock	ND (0/6)
Untreated HAV		127.77 ± 2.95 (6/6)	Untreated HAV	129.07 ± 1.37 (6/6)

37°C		ΔtCI_50_ (h)	65°C		ΔtCI_50_ (h)
Heat-treated HAV	2 days	15.34 ± 1.44 (6/6)	Heat-treated HAV	30 s	2.67 ± 1.33 (6/6)
	3 days	27.52 ± 1.17 (6/6)		1 min	11.71 ± 3.71 (6/6)
	4 days	49.16 ± 1.76 (6/6)		2 min	42.10 ± 3.26 (6/6)
	5 days	60.50 ± 2.46 (6/6)		5 min	64.50 ± 1.12 (2/6)
	7 days	68.82 ± 4.59 (5/6)		10 min	ND (0/6)
	9 days	ND (0/6)		20 min	ND (0/6)

50°C	tCI_50_ (h)	72°C	tCI_50_ (h)
Mock	ND (0/6)	Mock	ND (0/6)
Untreated HAV	125.22 ± 5.32 (6/6)	Untreated HAV	137.05 ± 0.80 (6/6)

50°C		ΔtCI_50_ (h)	72°C		ΔtCI_50_ (h)
Heat-treated HAV	1h	5.54 ± 0.93 (6/6)	Heat-treated HAV	30 s	2.38 ± 2.14 (6/6)
	2h	10.84 ± 1.89 (6/6)		1 min	24.72 ± 8.08 (6/6)
	4h	21.01 ± 2.41 (6/6)		2 min	74.16 ± 2.38 (2/6)
	6h	36.31 ± 2.54 (6/6)		5 min	ND (0/6)
	16h	41.65 ± 2.50 (2/6)		10 min	ND (0/6)
	24h	ND (0/6)		20 min	ND (0/6)

56°C	tCI_50_ (h)	Dynamic range	tCI_50_ (h)
Mock	ND (0/6)	Mock	ND (0/6)
Untreated HAV	131.47 ± 5.17 (6/6)	Untreated HAV	127.84 ± 1.64 (6/6)

56°C		ΔtCI_50_ (h)	Dynamic range		ΔtCI_50_ (h)
Heat-treated HAV	5 min	10.09 ± 2.49 (6/6)	Heat-treated HAV	30 s	4.29 ± 1.39 (6/6)
	15 min	13.47 ± 3.27 (6/6)		1 min	69.62 ± 2.27 (6/6)
	30 min	21.30 ± 3.57 (6/6)		2 min	ND (0/6)
	1 h 30 min	43.61 ± 9.64 (6/6)			
	2 h 15 min	56.69 ± 18.54 (5/6)			
	3 h	65.23 ± 6.25 (3/6)			

^*a*^ tCI_50_, the time to reach 50% reduction in impedance signal in HAV-infected cells, is calculated for every CI curve. ΔtCI_50_, the difference between the tCI_50_ of the HAV inoculum and the tCI_50_ of the heat-treated HAV sample. Data are presented as means (hours) ± s.e.m. obtained from three experiments performed in duplicate (*n* = 6). The number of tCI_50_ values determined for each condition is indicated in brackets (*n*/6: number (*n*) of samples with a tCI_50_ value out of 6 samples).

^*b*^ ND, no tCI_50_ value (CI remained above 50% of the CI_max_ throughout the experiment).

To assess viral reduction at each temperature and heat treatment duration, the delays in tCI_50_ for heat-treated HAV compared to the HAV inoculum are reported as ΔtCI_50_ values in Table 1. As previously observed with the standard curve, a ΔtCI_50_ delay of approximately 20 h corresponds to about a 1 log_10_ decrease in infectious HAV. The first 1 log_10_ reduction in infectious titers was reached after heat treatment of HAV during 2–3 days at 37°C, 4 h at 50°C, 30 min at 56°C, 1–2 min at 65°C, 1 min at 72°C, and less than 1 min at 80°C. The last temperature–time pairs at which infectious viruses were still detectable in all heat-treated samples were as follows: 37°C for 5 days, 50°C for 6 h, 56°C for 1 h 30 min, 65°C for 2 min, and 72°C for 1 min. Nevertheless, all these temperature–time pairs led to a decrease of the amount of infectious viruses, between 2 and 3 log_10_, except at 72°C during 1 min, leading to a 1 log_10_ decrease. No more infectious HAV could be detected by the RTCA assay after treatment at 37°C, 50°C, 65°C, 72°C, and 80°C lasting 9 days, 24 h, 10 min, 5 min, and 2 min, respectively.

Thus, the RTCA assay is a suitable method for quantifying the decrease in infectious HAV as the time–temperature scale increases.

### Thermal inactivation curves of HAV with different profiles depending on the titration method

**Fig 3:**
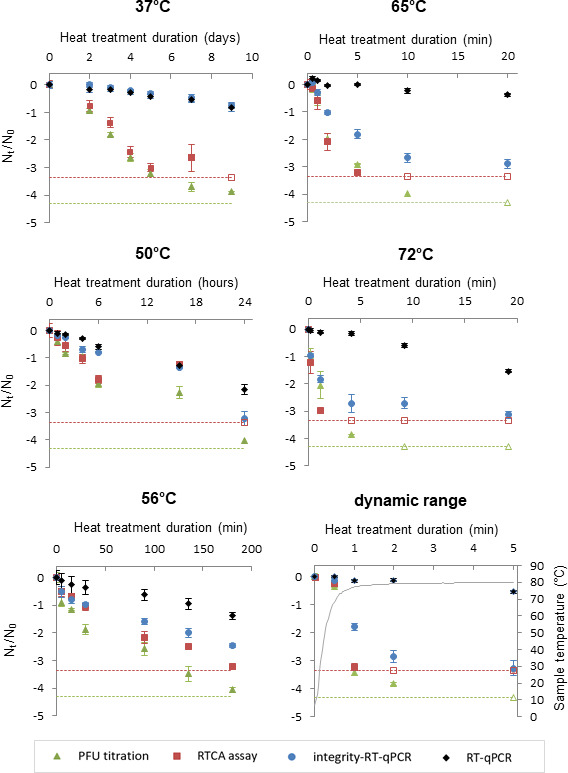
Thermal inactivation curves of HAV. The logarithm reduction of HAV (*N*_*t*/_*N*_0_) was plotted against the treatment time at each temperature. The limit of quantification of the RTCA assay and plaque assay is displayed as dotted lines, and the data under the limit of quantification are displayed as empty symbols.

To assess HAV behavior following heat treatment, HAV was quantified as viral genomes or infectious particles by using molecular-based methods (RT-qPCR and integrity-RT-qPCR) and cell culture-based methods (RTCA and Plaque assays), respectively. The reductions in the HAV concentrations were measured to establish the thermal inactivation kinetics of HAV for each heat treatment (Fig. 3).

At constant temperature, the HAV thermal inactivation kinetics followed different profiles according to the quantification method used. The decay curves obtained by the RTCA and PFU assays had the same profiles for every temperature, as both methods measured the reduction of infectious HAV following heat treatments. Nevertheless, for the highest duration tested at 37°C and 50°C, infectious HAV could not be quantified using the RTCA assay, as the HAV infectious titers were outside the quantification range of the standard curve. As both cell-culture methods have a different limit of quantification, the limits of the HAV reduction were estimated at −3.35 and −4.30 log_10_ infectious particles of HAV using the RTCA and plaque assays, respectively. On the contrary, only slight reductions in HAV genomic titers were obtained using the RT-qPCR assay throughout the duration of heat treatment. For the longest durations of heat treatments, the average of the genomic titers reductions ranged from 0.37 log_10_ (65°C; 20 min) to 2.17 log_10_ (50°C; 24 h). The persistence of viral genome, estimated by RT-qPCR, seems similar for any temperature tested, while the one estimated with integrity-RT-qPCR assay showed an impact of the highest temperatures on genomic titers (i.e., −2.89 log_10_ (65°C; 20 min) and −3.13 log_10_ (72°C; 19.2 min). Moreover, the kinetic profiles obtained by integrity-RT-qPCR were close to the ones obtained with both cell-based methods at 65°C and 72°C.

These results indicate that the kinetics of HAV inactivation following heat treatment present different profiles according to the method used, with integrity-RT-qPCR providing a better correlation with infectivity than RT-qPCR. Overall, the data highlight the importance of carefully selecting the quantification method to accurately assess the efficacy of an inactivation treatment.

### Model selection for HAV thermal inactivation curves

Different mathematical models were then applied to assess the one having the best fit with the experimental data from heat-inactivated HAV. The shapes of HAV thermal inactivation curves were generally characterized by an initial drop in viral titer followed by a tailing behavior; they seemed to fit a nonlinear model. Three inactivation models were evaluated (i.e., Bigelow, Geeraerd, and Weibull), and the Root Mean Sum of Squared Error (RMSE) was determined for each model at every temperature and for each titration method (Table 2). RMSE measures the average difference between experimental and predicted values, with lower values indicating better model accuracy. A two-way analysis of variance (ANOVA) was performed to assess the effects of model type and temperature on RMSE values (Fig. S1). For plaque assay data, both the Geeraerd and Weibull models fitted significantly better than the Bigelow model (*P* = 0.0475), while for RTCA data, the Geeraerd model showed a significantly better fit than the Bigelow (*P* = 0.0635). No significant differences between the models were observed for either of the molecular titration methods (integrity-RT-qPCR and RT-qPCR; *P* = 0.06). Temperature had a significant effect on RMSE values across all titration methods (*P* < 0.05).

**TABLE 2:** RMSE values obtained for each incubation temperature, detection method, and inactivation model with GInaFiT software

Detection method	Model	Incubation temperature (°C)*^a^*				
		37°C	50°C	56°C	65°C	72°C
Plaque assay	Bigelow	0.471	0.435	0.541	0.624	0.796
	Weibull	0.416	**0.355**	**0.415**	0.541	**0.681**
	Geeraerd	**0.252**	0.385	0.530	**0.441**	0.726
RTCA	Bigelow	0.568	0.688	0.467	0.424	**0.683**
	Weibull	0.575	0.621	**0.432**	0.406	0.704
	Geeraerd	**0.456**	**0.551**	0.453	**0.223**	0.704
Integrity RT-qPCR	Bigelow	0.225	0.353	0.410	0.603	0.836
	Weibull	**0.220**	**0.346**	**0.364**	0.430	**0.450**
	Geeraerd	0.223	0.361	0.407	**0.316**	0.489
RT-qPCR	Bigelow	**0.216**	**0.218**	0.543	**0.145**	0.162
	Weibull	0.217	0.220	0.536	0.146	**0.144**
	Geeraerd	0.219	0.224	**0.529**	**0.145**	0.165

^*a*^ The lowest RMSE value among the three models is indicated in bold for each detection method at every temperature.

Based on overall performance across all titration methods, the Geeraerd model was selected as the most appropriate for describing HAV thermal inactivation kinetics.

### HAV thermal resistance: method-dependent inactivation parameters

To determine the kinetic model parameters based on the experimental data of thermal inactivation of HAV, the best-fit model Geeraerd was used to calculate the *D* value at each temperature for every detection method (Table 3). The *D* values, representing the time required to achieve a 1 log_10_ reduction in HAV titer*,* were comparable between both cell-culture-based methods across all tested temperatures, ranging from 37.50 h at 37°C to 0.14 min at 72°C (RTCA assay) and 35.59 h at 37°C to 0.64 min at 72°C (Plaque assay). In contrast, molecular methods yielded significantly higher *D* values compared to cell-based assays, indicating overestimation of the time required for viral inactivation. For RT-qPCR, *D* values were 3.3- to 7.5-fold higher than plaque assay values at lower temperatures (37°C, 50°C, and 56°C), with this discrepancy increasing to 20-fold at higher temperatures (65°C and 72°C). The integrity-RT-qPCR method showed intermediate performance, with *D* values similar to those of cell-based assays only at the highest temperature (72°C: 0.73 min vs 0.64 min for the plaque assay). At intermediate temperatures, integrity-RT-qPCR overestimated inactivation times by factors of 7.67 (37°C), 2.46 (50°C), 1.59 (56°C), and 2.54 (65°C) compared to the plaque assay.

**TABLE 3:** *D* values obtained with GInaFiT software using HAV thermal inactivation data at 37°C, 50°C, 56°C, 65°C, and 72°C expressed with the Geeraerd model, according to the detection method

Heat treatment	RTCA assay	Plaque assay	Integrity-RT-qPCR	RT-qPCR
37°C	37.50 h	35.59 h	272.82 h	267.74 h
50°C	2.84 h	3.34 h	8.22 h	11.51 h
56°C	43.31 min	43.04 min	68.39 min	143.91 min
65°C	0.91 min	0.98 min	2.49 min	19.51 min
72°C	0.14 min	0.64 min	0.73 min	12.74 min

### Temperature-independent parameters: comparative analysis by detection method

Secondary model parameters (D₇₀ and *Z* values) were derived from isothermal data using linear regression at the reference temperature of 70°C (Table 4). The *D*₇₀ values confirmed the trends observed at individual temperatures, with RT-qPCR yielding significantly higher values (10.68 min) compared to other detection methods (0.33–0.98 min), highlighting the tendency to overestimate viral persistence when detecting total viral RNA versus viral RNA from intact capsids. The *Z* value represents the temperature increase required to achieve a tenfold reduction in the *D* value, and thus indicates the resistance of the virus to temperature changes. Notably, *Z* values showed consistency across all detection methods, ranging from 7.74°C to 10.86°C.

**TABLE 4:** *D* values at the reference temperature 70°C (*D*_70_) using the Geeraerd model and *Z* values obtained from the linear regression of the temperature of inactivation (*T*-70) against the logarithm of the *D* value for each titration method with Statgraphics

	RTCA assay	Plaque assay	Integrity-RT-qPCR	RT-qPCR
*D*_70_ value (min)*^a^*	0.33	0.79	0.98	10.68
*Z* value (**°**C)	8.04	9.34	7.74	10.86

^*a*^ *D*_70_ value, *D* value at the reference temperature 70°C.

### Dynamic temperature validation of the secondary model

**Fig 4:**
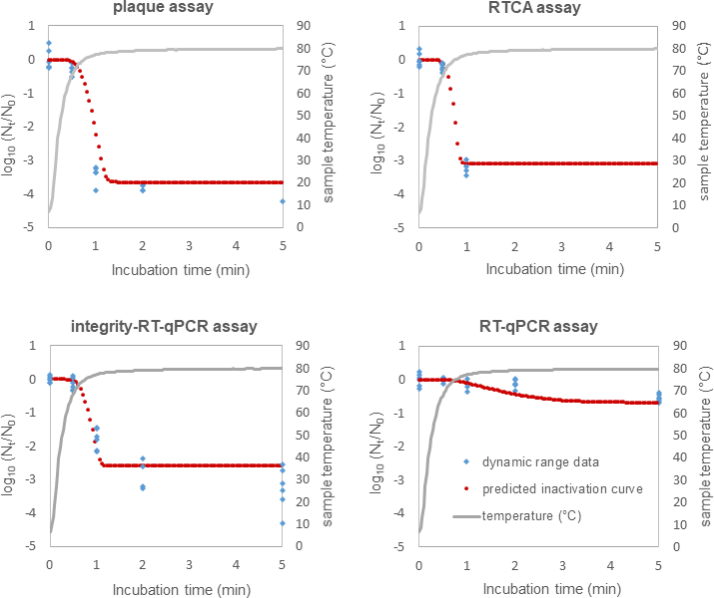
HAV inactivation curves at a dynamic range of temperature from 5°C to 80°C. The inactivation model Geeraerd was used to predict HAV inactivation curves with Bioinactivation software, using the inactivation kinetics parameters (*D* value at reference temperature 70°C, *Z* value, mean *N*_res_, and mean *N*_0_) determined with HAV thermal inactivation at 37°C, 50°C, 56°C, 65°C, and 72°C. In blue: experimental data set obtained under a dynamic temperature range. This independent data set was not included in the model development. In red: predicted inactivation curves.

To validate the secondary model parameters, an independent experimental data set obtained under dynamic temperature conditions (5°C–80°C) was used. The HAV thermal inactivation kinetics predicted using the derived parameters (*D*₇₀, *Z* value, mean *N*_res_, and *N*_₀_) for each detection method were compared to these experimental results (Fig. 4). This validation approach is essential in predictive microbiology, as it tests model performance on independent experimental data not used for parameter estimation, rather than relying solely on simulations. The use of dynamic temperature conditions further challenges the model since isothermal-derived parameters must accurately predict viral behavior under the fluctuating thermal conditions typical of industrial processes. The experimental data (in blue) showed good agreement with predicted inactivation curves (in red) across all titration methods, demonstrating the robustness of the Bigelow secondary model for HAV thermal inactivation. This successful validation confirms that the isothermal-derived parameters can reliably predict viral inactivation under dynamic thermal conditions.

## DISCUSSION

Our study on the impact of heat treatment on HAV persistence may enhance strategies for controlling virus contamination in food through thermal processing. Of the three nonlinear models tested, the Geeraerd model was the most accurate in describing isothermal HAV inactivation in a simple matrix, regardless of the quantification method used. Modeling inactivation curves enabled the prediction of the time required for effective HAV inactivation.

The titration method should be carefully selected to carry out viral inactivation studies. Indeed, HAV genomes tend to persist longer than infectious viruses, as widely reported (27–29). Viral genome detected by RT-qPCR can also be housed in a non-infectious viral particle, leading to false-positive results for infectivity (30, 31). Cell culture-based assays are the gold standard methods for inactivation studies, providing a direct measurement of viral infectivity. Nevertheless, for viruses that are difficult to cultivate (e.g., wild-type strains of HAV, human norovirus), alternative methods have been developed, such as the combined methods based on detection of viral genomes from intact viral particles (i.e., integrity-PCR assays) (23–25, 32, 33). As previously observed by us and others, integrity-RT-qPCR provided results consistent with infectious titration assays when viruses were heat-treated at high temperatures (24, 33, 34). High temperatures (>50°C) may induce sufficient denaturation of the viral capsid, allowing the penetration of integrity markers. In contrast, mild temperatures (<50°C) may cause only minor damage to the viral particles. This may lead to structural changes in the binding site of HAV with its cellular receptor, impairing the viral particle’s ability to attach to the cellular receptor. Nevertheless, most of the viral particles may remain intact enough to protect the viral genome, avoiding the penetration of the integrity marker and leading to an overestimation of the titer. A better understanding of the structural changes in the viral capsid following heat treatment could provide valuable insights into the mechanisms underlying HAV inactivation (35).

Although integrity-PCR assays have been widely developed for enteric viruses such as human norovirus, their application in testing field samples or conducting viral persistence studies remains limited. Even if integrity-PCR assays are one of the most promising methods for viruses that are difficult to cultivate and are preferable to using PCR alone, they still do not enable risk assessment (36). When a virus can be cultured, traditional cell-culture-based titration methods (i.e., PFU and TCID50 assays) should be used to quantify infectious viruses. To overcome their disadvantages (e.g., time-consuming, labor-intensive, subjective interpretation, higher variability), we also applied the RTCA assay for quantifying infectious HAV (26). The RTCA assay provided quantitative data that strongly correlated with those obtained using the conventional PFU method. The RTCA assay has a sensitivity of 1 PFU per well (100 µL), showing a dynamic range of up to 5 log_10_ PFU of HAV, leading us to set a lower quantification limit of 1 log_10_ PFU/100 µL based on the calibration curve. Regarding the PFU assay, we took into account up to one single plaque per well (corresponding to a virus titer of 1 PFU/mL), whereas other studies recommend a minimal plaque count for the plaque assay linear counting domain, from 5 to 20 plaques/well (37–40). In addition, in both assays, heat-treated samples were subjected to a further tenfold dilution prior to titration, which adds another factor of sensitivity loss. Consequently, the maximum observable reduction was limited to 4.30 log_10_ using the PFU assay, while a reduction of up to 3.35 log_10_ could theoretically be detected with the RTCA assay, which represents a limitation in our experimental design. However, one of the significant advantages of the RTCA method using the MP xCELLigence device is the ability to analyze a large number of samples. This high-throughput analytical capacity could also be crucial in other areas of virology, such as screening antiviral drugs.

Modeling data of heat inactivation of HAV is in line with previous studies underlining the non-linearity of the kinetics of viral inactivation. By comparing the performance of three different inactivation models using RMSE values, the Geeraerd model provided the best fit for every viral inactivation kinetics, regardless of the quantification method used. It allowed the comparison of the kinetic parameters obtained according to the analytical method used in a static temperature condition. *D* values obtained using PFU and RTCA assays were similar regardless of the heat treatment, whereas the use of RT-qPCR led to an increase in *D* values by a factor ranging from 3.5 to 20. *D* values obtained with data from the integrity-RT-qPCR assay were close to the ones obtained with quantitative data of infectious viruses for temperatures higher than 50°C (with a factor < 2.5). The overestimation of viral heat resistance using integrity-RT-qPCR appears to be a frequent observation, which can be assigned to the protection of the viral genome by the viral capsid. Indeed, some structural alteration of viral particles may be sufficient to inactivate their ability to enter host cells, while still preserving enough integrity to protect the viral genome. Our results are in line with Chen et al. (32), showing that integrity-RT-qPCR was unable to discriminate between native and heat-inactivated HAV below 72°C. At this temperature, we found a similar *D* value (e.g., 0.73 min by integrity-RT-qPCR *versus* 0.14 and 0.64 by RTCA and PFU, respectively). Similar *D*-values were also obtained by others at 72°C in culture medium (0.88–1.26 min) (41, 42). As a whole, our results showed that infectious particles of HAV have a great stability to temperature up to 50°C.

Nevertheless, heat treatment at high temperatures needs to take the temperature rise into account. Thus, to give a better estimation of parameters obtained under realistic dynamic environments, closer to real processing conditions at which the virus is exposed, the nonlinear parameters have been further based on dynamic (simulated) data of specific profiles (70°C). The reference temperature of 70°C is commonly used (43), and it is close to the upper limit of the dynamic range of temperature, 5°C–80°C. The use of a reference temperature allows the comparison of the heat resistance of HAV across different studies, even when experimental conditions vary. The simulated *D_70_* value predicted for the integrity-RT-qPCR was similar to the one predicted for cell culture-based assays (range 0.33–0.98 min), but strongly different from the *D_70_* value predicted using a genomic titration (10.68 min). The *Z* values (i.e., thermal resistance) predicted were in the same ranges regardless of the quantification method (from 7.74°C to 10.86°C), and close to the values previously reported in other studies (from 12.5°C to 14.8°C) using an infectious titration assay to quantify HAV in simple medium and in food (41, 44–46). Nevertheless, Coudray-Meunier et al. observed different *Z* values depending on the detection approach (PFU: 22°C; RT-qPCR: 44°C; integrity-RT-qPCR: 17°C) (24). In the same manner, the EFSA scientific opinion, based on PFU or TCID50 data, reported higher *Z* values around 28°C (95% CI: 14°C–41°C) (19). These discrepancies may be explained by differences in the temperature ranges used for *Z* value estimation. While the present study used a moderate temperature range (37°C–72°C), the EFSA analysis and other studies reporting higher *Z* values typically employed broader or higher temperature ranges (e.g., 55°C–100°C), which can influence the slope of the thermal inactivation curve and consequently affect *Z* value calculations (47). In our study, *Z* values showed consistency across all detection methods. This similarity suggests that the temperature dependence of HAV thermal inactivation may be an intrinsic viral characteristic, largely independent of the detection approach. These overall results highlight the need for conducting further studies across a wider range of temperatures to develop more robust and reliable thermal inactivation models.

When comparing HAV inactivation thermal kinetics among different studies, a wide variation is observed, depending on many parameters among which are the titration method, the matrix, and the inactivation model. The modeling of our HAV inactivation data by plaque assay and RTCA assay resulted in thermal inactivation kinetics in the same range to those obtained in other studies performed in simple medium (buffer, culture medium, or water), where *D* values ranged from 56 min to 12 h at 50°C, 1 min to 10 min at 65°C, and 0.88 min to 7.57 min at 72°C (41, 42, 48–50). Bozkurt et al. studied HAV thermal inactivation in various matrices. *D_60_* values obtained in complex food matrix (spinach, clam meat, blue mussel, turkey deli meat) were higher (from 3.25 min to 6.13 min) than in culture medium (2.67 min) (41, 44–46, 51, 52).

Viral inactivation studies on complex matrices (e.g., food, wastewater) remain challenging, largely because such samples may contain compounds that induce cytotoxicity. To mitigate these effects, eluates from complex matrices often need to be purified or diluted prior to being added to cell cultures, which reduces the amount of virus that can be detected. Together with the viral loss that occurs during extraction from complex matrices, these constraints reduce the sensitivity of traditional assays and narrow the quantifiable range of HAV (44, 45, 53, 54). Ongoing work in our laboratory is assessing whether impedance measurements can reliably quantify infectious virus directly from such matrices.

Future research should not only clarify the influence of detection methods on kinetic parameters but also investigate the influence of matrix factors (e.g., pH or sugar content) on HAV thermal resistance, as these factors are likely to affect viral stability and, consequently, inactivation kinetics and the resulting kinetic parameters. This study also provides valuable insights into HAV behavior under thermal conditions using a cell culture-adapted HAV strain. Nevertheless, these findings may not fully reflect the diversity of field strains encountered in real-world contamination events. A broader range of wild-type HAV isolates should be included in the framework of inactivation studies to enhance the accuracy of the predictive outcomes. Since wild types of HAV are difficult to cultivate, the integrity-RT-qPCR assays could help in providing these data. Overall, further studies would enable the development of more reliable and realistic models, leading to a better prediction of viral inactivation that can be applied across a variety of food processing.

In conclusion, our study demonstrated significant method-dependent differences in HAV thermal inactivation assessment and validated the use of RTCA assay to conduct viral inactivation studies. The development of the first predictive model for HAV inactivation offers an effective time-temperature scale to guide food safety strategies, and its validation under dynamic thermal conditions supports its applicability to industrial processes.

## MATERIALS AND METHODS

### Cell line and virus stocks

The FRhK-4 (fetal rhesus monkey kidney) cell line was purchased from the American Type Culture Collection (ATCC CRL-1688) (LGC standards SARL, Illkirch, France). These epithelial cells were cultured in Dulbecco’s modified Eagle’s medium—Glutamax (DMEM) supplemented with non-essential amino acids and 10% of heat-inactivated fetal bovine serum (Thermo Fisher Scientific, Waltham, MA, USA). Cells were maintained at 37°C in a humidified atmosphere containing 95% air and 5% CO_2_.

The cell culture-adapted HM175-18f strain of HAV was obtained from ATCC (ATCC VR1402). This clone replicates rapidly and has cytopathic effects in cell culture (55, 56). HAV stock was produced by propagation in FRhK-4 cells and titrated by lysis plaque assay (57). Results were expressed in PFU/mL, and the HAV stock contained 10^7^ PFU/mL. The supernatant was aliquoted and stored at −80°C.

### Heat treatment of HAV

HAV stocks were fivefold diluted in DMEM and aliquoted in Safe-Lock 1.5 mL microtubes (Eppendorf, France). Diluted viral suspensions of HAV (2 × 10^5^ PFU/100 µL) were held at 5°C (native virus) or incubated (heat-treated virus) at 37°C in an incubator or at 50°C, 56°C, 65°C, 72°C, or 80°C in a dry bath (Thermostat Plus, Eppendorf, France). The temperature of samples was measured with a DAQSTATION DX100 recorder (Yokogawa, Vélizy Villacoublay, France). At the end of the heat treatment, samples were immediately placed on ice and 10-fold diluted in cold PBS. Four aliquots of each sample (inoculum or heat treated) were prepared and stored at −80°C for later HAV quantification by using the following methods: plaque assay, RTCA assay, RT-qPCR, and integrity-RT-qPCR. Each set of experiments was performed three times.

### HAV titration by plaque assay

HAV titration by plaque assay was performed as previously described (57). FRhK-4 cell monolayers were grown in six-well culture plates at 37°C under 5% CO_2_. Cells were washed once with serum-free medium and 30 min later inoculated with 500 µL per well of inoculum or heat-treated samples 10-fold diluted in DMEM. After virus adsorption for 2 h at 37°C, 3 mL per well of semi-solid overlay consisting of M199 supplemented with 0.56% agarose, 12% fetal bovine serum, non-essential amino acids, glutamine, sodium pyruvate, and 0.5% penicillin/streptomycin (Thermo Fisher Scientific) were added to each well. At 5 days post-infection, 1.5 mL per well of semi-solid overlay was added. At 11 days post-infection, cell monolayers were fixed in a solution of 0.2 N HCl for 20 min, the agarose overlay was removed, and the cells were stained with 0.1% crystal violet solution for plaque visualization (Sigma-Aldrich, Saint-Quentin Fallavier, France) to reveal plaques, which were counted.

### HAV titration by RTCA assay

To carry out HAV titration by RTCA assay, the xCELLigence DP system (Agilent, Les Ulis, France) was used according to the manufacturer’s instructions.

This RTCA system comprises four main components: the RTCA DP Station with three independent E16-well plate platforms placed inside a tissue-culture incubator; the RTCA sensor Analyzer for sending and receiving the electronic cellular signals; the RTCA computer (Control Unit) with integrated software (RTCA Software 2.0, ACEA Biosciences) to acquire and display data in real time; and the disposable E-Plates 16. The E-plate is a standard 16-well plate with a glass bottom coated with gold microelectrodes covering approximately 75% of the well area.

The xCELLigence system measures changes in impedance and calculates them as a dimensionless parameter called Cell Index: CI = (*Z*_i_ − *Z*_0_)/15. *Z*_i_ is the impedance at an individual point of the experiment, whereas *Z*_0_ describes the background measurement at the start of the experiment. CI is derived from the impedance changes at 10 kHz frequency.

Measured electrical impedance is translated as a dimensionless parameter, the CI. When there are no cells on the electrode surface, CI value is zero, corresponding to the background linked to the free flowing of ions in the cell culture medium. In contrast, CI values increase progressively and proportionally as cells attach to the electrodes. The CI variations are displayed in a real-time plot by the software. The data generated with the integrated software were exported to Excel.

The RTCA assay was performed as previously described (26). First, 100 µL of cell culture medium (DMEM supplemented with 10% FBS) was added to each well of the E-plate 16. The E-plate 16 was then connected to the system to check in the cell culture incubator for proper electrical contacts and to obtain background impedance readings in the absence of cells. Meanwhile, FRhK-4 cells were seeded onto the E-plate at a density of 10,000 cells per well in 100 µL of cell culture medium. E-plate 16 was placed onto the RTCA DP Station located inside the incubator (5% CO2; at 37°C) for continuous impedance recording. CI values were measured every minute for 4 h and then every 15 min. At 2 days post-seeding, cell monolayers were washed once with serum-free medium and 30 min later infected with 100 µL per well of inoculum or heat-treated samples. Following the virus adsorption lasting 2 h at 37°C, the inoculum was kept, and 100 µL per well of culture medium supplemented with 4% FBS was added. In parallel, mock-infected cells were treated exactly in the same way.

To quantify HAV by RTCA assay, the time taken for 50% decrease of the CI_max_ (tCI_50_) was regressed as a linear function of the log_10_ of the HAV inoculum concentration (PFU/100 µL). HAV titer can be expressed as follows:


$$log_{10}\left(HAV\right)=\frac{\left(log_{10}\left({tCI}_{50}\right)-217.2\right)}{-20.12}$$

### RT-qPCR assay

Samples were supplemented with NucliSens easyMAG lysis buffer (BioMérieux, Marcy-l'Étoile, France) up to 3 mL and subjected to the NucliSens easyMAG platform for total nucleic acid extraction by the “off-board Specific A protocol,” according to the manufacturer’s instructions. Nucleic acids were eluted in 70 μL of elution buffer and stored at −80°C. The primers and probe (Eurofins Genomics, Les Ulis, France) targeting the non-coding region at the 5′ end of HAV and generating an amplification product of 353 bp were described by Coudray-Meunier et al. (24). One-step RT-qPCR amplifications were performed in duplicate on RNA extracts on a CFX96 real-time PCR detection system from Bio-Rad (Marnes-la-Coquette, France) as previously described (24). A HAV *in vitro* transcribed (IVT) RNA standard curve was included in duplicate on each RT-qPCR plate. HAV IVT RNA was quantified using a linearized plasmid DNA standard curve (58) and serially diluted tenfold in nuclease-free water (1 × 10^2^ to 1 × 10^6^ genome equivalents per µL).

### Integrity-RT-qPCR assay

EMA (phenanthridium, 3-amino-8-azido-5-ethyl-6-phenyl bromide) (Thermo Fisher Scientific) was dissolved in absolute ethanol to create the stock concentration of 5 mg/mL and then dissolved in ultrapure RNAse-free water to obtain a 400 µM solution stored at −20°C in the dark. Experiments were performed in light-transparent 1.5 mL microcentrifuge tubes. IGEPAL CA-630 (Sigma-Aldrich) was diluted in ultrapure RNAse-free water to obtain a 10% stock solution. Five microliters of EMA and IGEPAL CA-630 were added to each sample to have a final concentration of 20 μM and 0.5%, respectively. Samples were incubated for 2h at 4°C in the dark and then exposed to light for 15 min using the LED-Active Blue system. After photo-activation, samples were subjected to nucleic acid extraction. Finally, one-step RT-qPCR amplifications were performed in duplicate on RNA extracts in the same manner as the RT-qPCR assay (24).

### Viral inactivation data and model validation

Log reduction values were calculated as log₁₀(*N*_0_/*N*_t_), where *N*_0_ represents the viral titer before treatment and *N*_t_ the titer after thermal treatment at time t. Data were reported as the mean ± the standard error of the mean (SEM).

#### Kinetic model fitting

GInaFiT (version 1.6), a freeware add-in for Microsoft Excel developed by Geeraerd et al. (59) for testing different types of microbial survival models, was used for modeling inactivation kinetics at 37°C, 50°C, 56°C, 65°C, and 72°C. Three models were tested and compared on heat-inactivated HAV data: the log-linear “Bigelow” model (60), the log-linear + tail “Geeraerd” model (61), and the “Weibull” model (62). GinaFit software provides model parameters and their corresponding standard error values.

The Bigelow model can be expressed as follows: $\log_{10}\left(N_{t}\right)=\log_{10}\left(N_{0}\right)-\frac{k_{max}t}{\ln\left(10\right)}$ , where *k*_max_ (min^−1^) and N_0_ are the model parameters. *k*_max_ is the first-order inactivation rate constant, that is, it characterizes the slope of the linear decrease of concentration expressed as a logarithm. *k*_max_ is directly linked to the *D* value (decimal reduction time): $k_{max}=\frac{\ln\left(10\right)}{D}$.

The Weibull model can be expressed as follows: $\log_{10}\left(N_{t}\right)=\log_{10}{(N}_{0})-{\left(\frac{t}{\delta }\right)}^{p}$ where *δ* (min)*, p,* and *N*_0_ are the model parameters. δ can be denoted as the time to first log-reduction if *p* = 1, and *p* is a shape parameter. For *p* > 1, convex curves are obtained, while for *p* < 1, concave curves are described.

The Geeraerd model can be expressed as follows: $\log_{10}\left(N_{t}\right)=\log_{10}\left[\left(10^{\log_{10}\left(N_{0}\right)}-10^{\log_{10}\left(N_{res}\right)}\right)\cdot e^{-k_{max}t}+10^{\log_{10}\left(N_{res}\right)}\right]$, where *k*_max_ (min^−1^), *N*_res_, and *N*_0_ are the model parameters. *N*_res_ is the starting point of the tail, that is, the residual viral concentration at the end of the decrease, not undergoing any significant subsequent inactivation regardless of the duration of the inactivation treatment. A tail is unlikely when the estimated value of *N*_res_ is lower than the smallest measured virus titer.

The RMSE determines the goodness of fit for models via the difference between predicted and observed values (best fit indicated when this value is close to zero). A two-way ANOVA was performed with the Statgraphics Centurion XVII software (version 17.1.04) on RMSE values obtained for each heat treatment and detection method, to test (1) the effect of the titration method and (2) the effect of the temperature. The result of the ANOVA is a *P*-value associated with the hypothesis that the means of all groups were the same. When the means were statistically different (ANOVA, *P* < 0.05), a multiple comparison procedure (Fisher’s least significant difference method) was applied to determine which condition was significantly different from the others. Graphs plotting the mean and its confidence interval for each group illustrate the multiple comparison procedure. The most appropriate inactivation model for describing HAV inactivation curves, regardless of the titration method (Geeraerd model), was selected for subsequent data analysis.

#### Temperature-dependent secondary modeling and validation

Linear regressions were performed on the temperature of inactivation (*T*-70) against the logarithm of the *D* value for each titration method using Statgraphics to establish Bigelow secondary models describing the temperature dependence of thermal resistance. The Bigelow secondary model, which assumes a log-linear relationship between *D* values and temperature, was applied to calculate the resulting kinetic parameters as follows: $D_{70}=10^{intercept}$; $Z=\frac{-1}{slope}$, where the *D*_70_ is the *D* value at 70°C; and the *Z* value represents the temperature change required for a one-log reduction in the *D* value. These Bigelow secondary models characterize the relationship between temperature and the primary inactivation parameters (*D* values) derived from isothermal conditions.

The secondary model parameters (*D*₇₀ and *Z* values) estimated from isothermal data sets (37°C, 50°C, 56°C, 65°C, and 72°C) were validated using dynamic temperature conditions (5°C–80°C). Predictive modeling was performed using the “Bioinactivation” web application (available at https://foodlab-upct.shinyapps.io/bioinactivation4/, last accessed December 17, 2025) (63) with the following parameters: Geeraerd as inactivation model, 70°C as reference temperature, *D* values at 70°C, *Z* values, mean *N*_0_, and *N*_res_ values obtained by each titration method, and no shoulder behavior. The application integrates primary and secondary models to predict inactivation under dynamic conditions, solving differential equations using the LSODA algorithm (64) via the deSolve R package (65), and generates plots comparing the predicted survivor curve and the experimental data. Temperature values at intermediate time points are calculated using linear interpolation between measured values.

## SUPPLEMENTAL MATERIAL

Fig. S1 aem.01822-25-s0001.docx RMSE means with 95% confidence intervals.

ASM does not own the copyrights to Supplemental Material that may be linked to, or accessed through, an article. The authors have granted ASM a non-exclusive, world-wide license to publish the Supplemental Material files. Please contact the corresponding author directly for reuse.
