# Supplementary material for: An RTCA-based assay as an innovative approach for thermal inactivation studies of hepatitis A virus
Source: Appl Environ Microbiol. 2026 Feb 10;92(3):e01822-25. doi: 10.1128/aem.01822-25 (PMC12997763; doi:10.1128/aem.01822-25)
Supplement: Fig. S1 — RMSE means with 95% confidence intervals. [file aem.01822-25-s0001.docx]

**
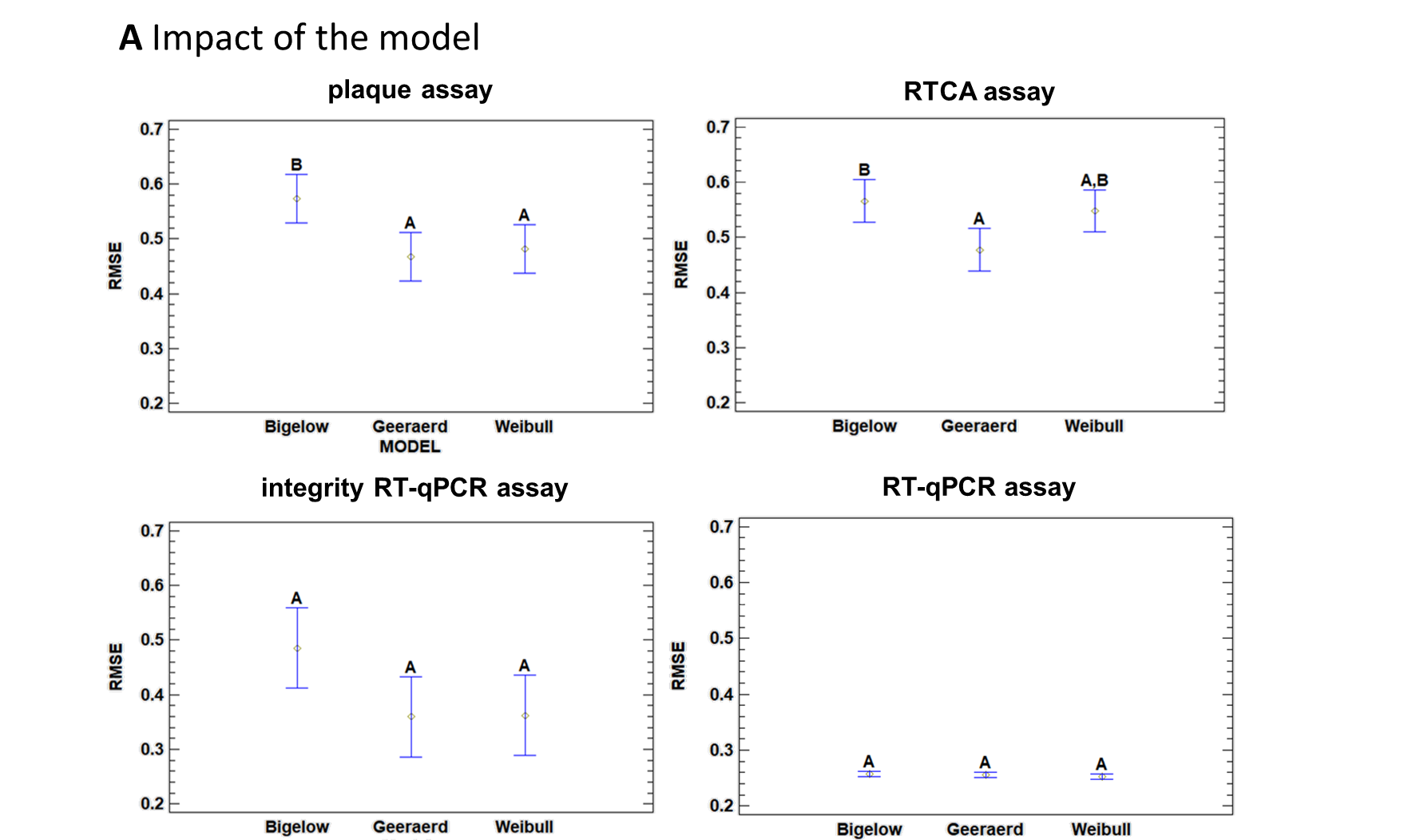
**

**
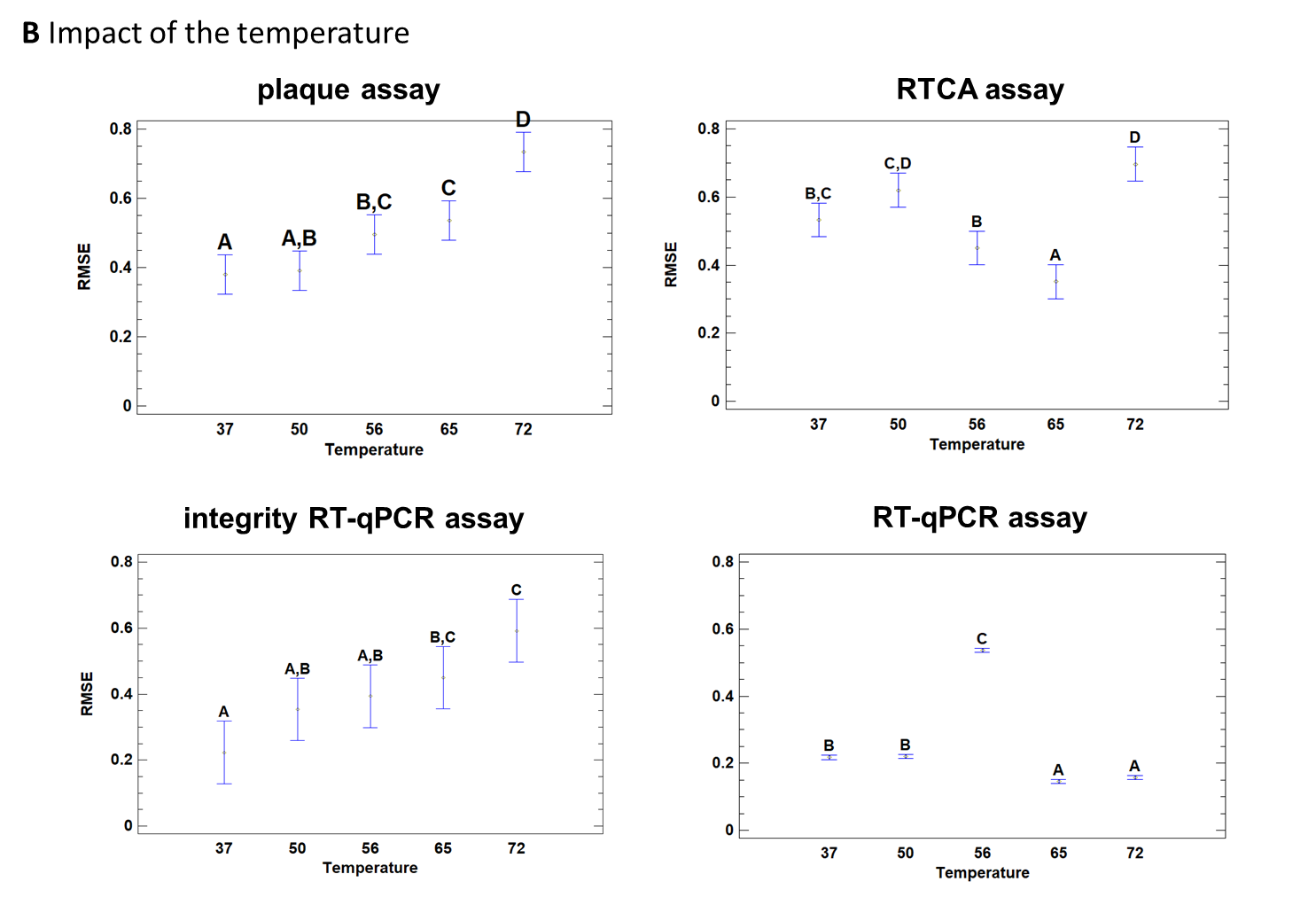
**

**Supplementary figure 1: RMSE means with 95% confidence intervals**. **A:** RMSE means according to the inactivation model (Bigelow, Geeraerd or Weibull). **B:** RMSE means according to the temperature. Two means are significantly different if their intervals are disjoint and are not significantly different if their intervals overlap. The letter above the confidence intervals indicates if the intervals are disjoint or not. The same letter indicates that the intervals overlap; different letters indicate that the intervals are disjoint.
